# SpaMFG: a spatial multi-omics integration method based on feature grouping

**DOI:** 10.1093/bioinformatics/btag457

**Published:** 2026-06-30

**Authors:** Zilin Li, Litian Ma, Jingtao Liu, Wei Sun, Yan Li, Chenguang Zhao, Liang Yu

**Affiliations:** School of Computer Science and Technology, Xidian University, Xi’an, Shaanxi 710126, China; School of Computer Science and Technology, Xidian University, Xi’an, Shaanxi 710126, China; School of Computer Science and Technology, Xidian University, Xi’an, Shaanxi 710126, China; Department of Rehabilitation Medicine, Xijing Hospital, Fourth Military Medical University, Xi’an, Shaanxi 710032, China; School of Management, Xi’an Polytechnic University, Xi’an, Shaanxi 710048, China; Department of Rehabilitation Medicine, Xijing Hospital, Fourth Military Medical University, Xi’an, Shaanxi 710032, China; School of Computer Science and Technology, Xidian University, Xi’an, Shaanxi 710126, China

## Abstract

**Motivation:**

The rapid development of spatial multi-omics technology enables the simultaneous measurement of gene and protein expression alongside spatial location, providing valuable insights into tissue heterogeneity. However, challenges such as low spatial resolution and high feature dimensionality complicate data integration and biological interpretation.

**Results:**

To address these issues, we propose SpaMFG, an innovative feature-group-level framework for interpretable spatial multi-omics integration. SpaMFG leverages spatial location information and introduces a spatial proximity weighting method to improve feature grouping accuracy. Additionally, it employs a new cross-omics feature group matching method that combines spatial location and Jaccard similarity to construct a weighted cost matrix, which is optimized using the Hungarian algorithm. This approach enhances the biological interpretability of cross-omics feature relationships. We evaluated SpaMFG’s performance through comparative analysis on the human lymph node dataset, demonstrating its effectiveness. Further applications on human tonsils, mouse spleens, and mouse thymus datasets confirmed the robustness of SpaMFG in various biological contexts.

**Availability and implementation:**

The source code for SpaMFG is available at https://github.com/LiangYu-Xidian/SpaMFG.

## 1 Introduction

In recent years, the emergence of spatial multi-omics sequencing technology has marked a significant transformation in the study of complex biological systems (Vandereyken *et al.* 2023, Ren *et al.* 2024). This technology captures multi-level molecular data such as gene expression and epigenetic modifications *in situ* and retains spatial location information, thereby revealing the molecular basis of intercellular interaction patterns and functional units of tissues. Representative methods include, spatial-CITE-seq (Liu *et al.* 2023), SPOTS (Ben-Chetrit *et al.* 2023), SM-Omics (Vickovic *et al.* 2022), Stereo-CITE-seq (Liao *et al.* 2023), and the Xenium from 10× Genomics (Janesick *et al.* 2023). Therefore, how to efficiently integrate spatial multi-omics data and accurately interpret their spatial-molecular associations has become an important challenge in this field.

Currently, there are relatively few studies on the integration methods of spatial multi-omics data. Thus, the representative methods SpatialGlue (Long *et al.* 2024) and MISO (Coleman *et al.* 2025) are selected for analysis. SpatialGlue is a spatial multi-omics data integration method based on a deep dual attention mechanism, aiming to fully integrate spatial topological structure and multi-omics feature information to achieve the analysis of tissue molecular heterogeneity. However, this method demands high computational resources and, due to its adoption of a deep learning architecture (Zulfiqar *et al.* 2023, Zhang *et al.* 2025), the interpretability of the model is relatively low. Although the attention mechanism provides some degree of interpretability, it still belongs to a “black box” model, making it difficult to intuitively understand its decision-making process and the biological basis behind it (Wei *et al.* 2017, Guo *et al.* 2024, Huang *et al.* 2024, Wang *et al.* 2024, Xie *et al.* 2025). MISO is another spatial multi-omics data integration method, aiming to reveal the biological characteristics and spatial structure of tissues. However, this method is rather limited in the modeling of spatial information, which may result in the failure to fully integrate regions that are physically close but have significant differences in genomic characteristics (Dai *et al.* 2022). As a consequence, the accuracy of the analysis is compromised.

Therefore, we propose SpaMFG, an innovative feature-group-level framework for interpretable spatial multi-omics integration. The core idea of SpaMFG is to identify transcriptomic and proteomic feature groups that exhibit similar spatial distribution patterns across tissue spots. First, SpaMFG groups genes and proteins into spatially coherent feature modules according to their spatially weighted expression patterns. Second, SpaMFG explicitly matches transcriptomic and proteomic feature groups by combining spot-neighborhood overlap and spatial distribution information. Third, the matched feature-group pairs are used as structured inputs for MOFA-based integration, generating latent representations for downstream spatial domain identification. Compared with SpatialGlue and MISO, SpaMFG emphasizes explicit discovery of spatially co-distributed transcriptomic–proteomic feature groups rather than only learning integrated latent embeddings. Compared with scMFG, SpaMFG extends feature grouping to spatial multi-omics by incorporating spatial proximity weighting and spatial-distribution-aware matching. To evaluate the performance of the SpaMFG model, we conducted a comparative analysis with existing methods on the human lymph node dataset, verifying the effectiveness of SpaMFG and applying it to human tonsils, mouse spleens, and mouse thymus datasets to further validate the robustness of the model.

## 2 Materials and methods

### 2.1 Data sources

#### 2.1.1 Data collection

We collected four datasets from two publicly available databases, namely the Gene Expression Omnibus and 10× Genomics databases. The four spatial multi-omics datasets used are the human lymph node dataset from 10× Genomics, the human tonsil dataset, the mouse spleen dataset, and the mouse thymus dataset processed by Liao *et al*. (2023). To ensure the quality of the input data for the model, quality control was performed on these four datasets. For the human lymph node dataset, low-quality spots with fewer than 200 expressed genes were filtered, as well as genes with fewer than 200 expressed spots. Feature selection in proteomics was not performed. For the human tonsil and mouse spleen datasets, low-quality spots with fewer than 10 expressed genes were filtered, as well as genes with fewer than 20 expressed spots. For the mouse thymus dataset, low-quality spots with fewer than 3 expressed genes were filtered, as well as genes with fewer than 20 expressed spots. In terms of data preprocessing, we preprocessed the transcriptomic and proteomic data using the standard workflow in the scanpy package (Wolf *et al.* 2018). This process included data normalization, logarithmic transformation, and feature selection steps. When screening highly variable genes, the top 4000 genes were selected for subsequent analysis. For the proteome data, the same preprocessing flow as for the transcriptome data was adopted. Due to the relatively small number of features in proteomics, no feature selection was performed for proteomics features.

### 2.2 Methods

**Figure 1:**
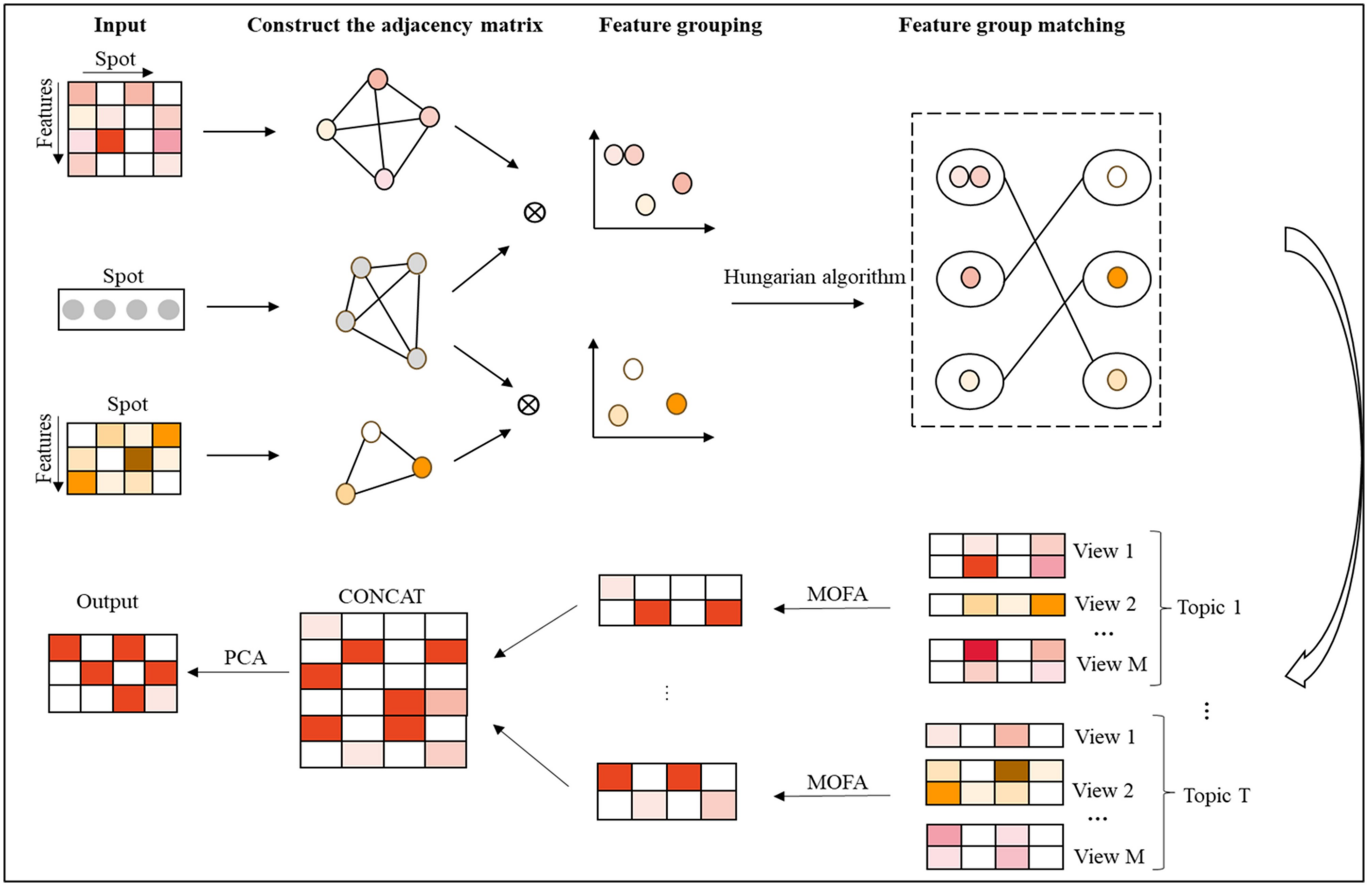
Overview of the SpaMFG framework for spatial multi-omics integration.

The spatial multi-omics data are integrated using the feature grouping method based on spectral clustering. The SpaMFG model mainly consists of three steps: (i) Identify the feature groups with similar spatial expression patterns in each omics; (ii) Analyze the shared spot expression patterns within each feature group and match the feature groups of different omics; (iii) Integrate the feature groups of different omics (Ma *et al.* 2025). The overall workflow of SpaMFG is shown in Fig. 1. These steps extend the feature-grouping idea of scMFG to spatial multi-omics data by explicitly incorporating spatial information.

#### 2.2.1 Feature grouping based on spectral clustering

To address the issues of noise and differences in molecular dimensions in spatial multi-omics data, and to more accurately identify the spatial domain, we adopt the feature grouping method of spectral clustering. Spectral clustering is a graph-based clustering method and is widely used in various fields such as single-cell analysis (Von Luxburg 2007, Liu *et al.* 2019, Li *et al.* 2021, Wei *et al.* 2021, Xu *et al.* 2023, Wang *et al.* 2023, 2025). By using spectral clustering, it is possible to effectively distinguish and separate features with similar spatial expression patterns. Feature grouping summarizes genes or proteins with similar spatial patterns into coherent modules, which reduces feature-level noise and provides more stable inputs for downstream spot-level spatial domain identification. Specifically, in SpaMFG, first, the expression matrix of the *m*-th omics layer is denoted as $Y_{m}\in R^{V\times N_{m}}$, where V is the number of spots and $n_{m}$ is the number of features in this omics layer. For features i and j, $Y_{m}^{i}$ and $Y_{m}^{j}$ denote their expression vectors across all spots, respectively. And the cosine similarity is calculated as follows:


(1) Sexpm(i,j)=Ymi.Ymj||Ymi||||Ymj||

After calculating the similarity matrix $S_{\exp}^{m}$ for all features in the m-th omics layer, the similarity between spots is then calculated based on the spatial coordinate information of the spots. Nearby spots often share local tissue microenvironmental signals; therefore, spatial weighting helps enhance coherent spatial expression patterns and reduce isolated noise. Let $X_{k}$ and $X_{l}$ denote the spatial coordinates of spots k and l, respectively. Spatial correlation between spot *k* and *l* is given by:


(2) Sspatial(k,l)=exp⁡(-||Xk-Xl||22σ2)

Among them, ${\left||X_{k}-X_{l}|\right|}^{2}$ represents the Euclidean distance between spatial spots *k* and *l*, and σ controls the spatial scale of the Gaussian kernel. We used σ=0.5 as a default setting across datasets to introduce local spatial smoothing without dataset-specific tuning.

Subsequently, the spatial similarity $S_{\mathrm{spatial}}(k,l)$ is multiplied by the original expression data $Y_{m}$ to generate the weighted expression data $\tilde{Y_{m}}$. The expression vectors of features i and j in ${\tilde{Y}}_{m}\;$are denoted as ${\tilde{Y}}_{m}^{i}$ and ${\tilde{Y}}_{m}^{j}$, respectively. The cosine similarity $S_{\mathrm{weight}\_\exp}^{m}$ between the features of the weighted expression data $\tilde{Y_{m}}$ is calculated:


(3) Sweightexpm(i,j)=Ymi∼.Ymj∼||Ymi∼||||Ymj∼||

Then, combine the weighted expression similarity $S_{\mathrm{weight}\_\exp}^{m}$ and the similarity matrix $S_{\mathrm{weight}\_\exp}^{m}$ to generate the final comprehensive similarity matrix S(i,j):


(4)  S(i,j)=α·Sweight_expm(i,j)+(1-α)·Sexpm(i,j)

Herein, α balances the original expression similarity $S_{\exp}^{m}$ and the spatially weighted expression similarity $S_{\mathrm{weight}\_\exp}^{m}$. We set α=0.5 by default to give equal weight to molecular expression similarity and spatially weighted expression similarity.

Subsequently, S(i,j) is taken as the input for spectral clustering. Through spectral clustering, the initial class labels are generated. Because spectral clustering may generate empty or highly imbalanced groups, we applied a post-clustering adjustment strategy. Each group was required to contain features within a predefined range [${FN}_{\min},\;FN_{\max}$], where $FN_{\min}$and $FN_{\max}$ denote the minimum and maximum numbers of features allowed in each group, respectively. Empty groups were skipped and excluded from subsequent matching and integration. For small non-empty groups with fewer than $FN_{\min}\;$features, candidate features were selected from the current largest group. For each candidate feature i, the target group was determined by a score that combines feature similarity and group-size balance:


(5) score(i,c)=S(i,C)

Here, S(i,C) represents the similarity between feature *i* and the features within category C, and the scores are sorted. For small categories with feature numbers less than ${FN}_{\max}$ but greater than or equal to ${FN}_{\min}$, an attempt is made to balance the category by adjusting the spatial consistency and the number of features dynamically. For each small category, the number of features needed to be supplemented is calculated as need:


(6) need=FNmin-labelcounts[c]

Here, $\mathrm{labe}l_{\mathrm{counts}[c]}$ represents the number of features in group c. Then, select appropriate candidate genes from other categories, where these features come from the categories with more than ${FN}_{\min}$ features. Achieve a balance between spatial consistency and feature number by combining them:


(7) score(i,c)=α1C∑j∈CS(i,j)+γ(FNmax-label_counts[c])

Among them, $\frac{1}{C}\sum_{j\in C}S(i,j)$ represents the spatial similarity between feature i and all features in the small category c. ${FN}_{\max}-\mathrm{labe}l_{\mathrm{counts}[c]}$ indicates the number of features that the target category still needs to acquire. In each iteration, the algorithm first assesses the current number of features for each category and compares it with the target allocation. Then, according to the set adjustment strategy, it prioritizes increasing or decreasing the categories with insufficient or excessive features to gradually approach category balance. The adjustment process will continue until the number of features for all categories meets the set threshold or reaches the maximum number of iterations to prevent infinite loops or waste of computing resources.

#### 2.2.2 Feature group matching based on the Hungarian algorithm

To match feature groups across different omics layers, the problem needs to be transformed into an indirect matching method of calculating the number of spot neighbors, in order to maximize the functional correlation between feature groups and minimize the spatial distribution differences. Specifically, for the transcriptome, the input *T* feature groups are:


(8) GRNA={G1RNA,G2RNA,…,GTRNA}

Among them, $G_{i}^{\mathrm{RNA}}$ denotes the i-th transcriptomic feature group. Similarly, the proteomic feature groups are:


(9) GProtein={G1Protein,G2Protein,…,GTRProtein}

where $G_{j}^{\mathrm{Protein}}$denotes the j-th proteomic feature group.

The objective of SpaMFG is to optimize the matching function $\varphi \cdot G^{\mathrm{RNA}}\to G^{\mathrm{Protein}}$ and identify the one-to-one correspondence between feature groups. The matching is performed at the feature-group level rather than at the individual gene/protein level. All retained transcriptomic features are first grouped into spatially coherent feature groups, instead of being randomly selected for matching. In the current implementation, both omics layers are grouped into the same number of feature groups before matching. This design provides clear cross-omics group pairs, but does not imply strict one-to-one biological regulation between genes and proteins. Therefore, a cost matrix $\mathrm{Cost}\in R^{T\times T}$ is defined, where the element ${\mathrm{Cost}}_{ij}$ represents the matching cost between the transcriptomic feature group $G_{i}^{\mathrm{RNA}}$ and the proteomic feature group $G_{j}^{\mathrm{Protein}}$. To calculate the cost matrix Cost, the degree of spot overlap between the two feature groups $G_{i}^{\mathrm{RNA}}$ and $G_{j}^{\mathrm{Protein}}$ needs to be computed based on the Jaccard similarity (Jaccard 1901).


(10) J(Smi,Snj)=|SmGiRNA∩SnGjProtein||SmGiRNA∪SnGjProtein|

Here, $S_{m}^{i}$ and $S_{n}^{j}$ denote the KNN spot sets constructed from the transcriptomic feature group $G_{i}^{\mathrm{RNA}}$ and the proteomic feature group $G_{j}^{\mathrm{Protein}}$, respectively. For each feature group, KNN is computed among spots using the spot-level expression profiles restricted to the features in that group. Spatial coordinates are not used in this KNN construction, but are incorporated separately through the spatial center distance term. Next, calculate the Euclidean distance between the spatial center points of the feature groups:


(11) Dspatial(GiRNA,GjProtein)=||ciRNA-cjProtein||2

where $C_{i}^{\mathrm{RNA}}$and $C_{j}^{\mathrm{Protein}}$denote the spatial centers of $G_{i}^{\mathrm{RNA}}$and $G_{j}^{\mathrm{Protein}}$, respectively.

For $G_{i}^{\mathrm{RNA}}$ and $G_{j}^{\mathrm{Protein}}$, the cost matrix Cost is constructed by combining the Jaccard similarity $J(S_{m}^{i},S_{n}^{j})$of their KNN spot sets and the spatial-center distance $D_{\mathrm{spatial}}(G_{i}^{\mathrm{RNA}},G_{j}^{\mathrm{Protein}})$ through a weighted approach:


(12)  Costij=-(α·J(Smi,Snj)+(1-α)·Dspatial(GiRNA,GjProtein))

Here, α controls the relative contribution of spot-neighborhood overlap and spatial-center distance.

Then, the maximum matching problem is transformed into a minimum matching cost problem and solved by the Hungarian algorithm (Kuhn 1956) to obtain the optimal mapping:


(13) $$\min\sum_{i=1}^{T}{\mathrm{Cost}}_{i,\varphi (i)}$$

Because the proteomic layer contains fewer measured features, some transcriptomic spatial patterns may only have weak protein-level support. Therefore, matched pairs are interpreted as the best available spatial correspondences under the measured omics features.

After obtaining the optimal matching index pair i,φ(i), each transcriptomic–proteomic feature-group pair with similar spatial distribution is treated as two omics views and integrated using MOFA (Argelaguet *et al.* 2018). MOFA is suitable for this step because it is an unsupervised multi-view factor model that learns latent factors from multiple omics views. MOFA learns shared latent factors for each matched pair, and the latent factors from all matched pairs are concatenated and further reduced by PCA to generate the final low-dimensional representation for downstream analysis. These matched groups and latent dimensions provide two levels of interpretability: traceable cross-omics group associations and latent factors associated with marker-protein spatial patterns.

## 3 Results

### 3.1 Validation of the effectiveness of feature grouping method based on spectral clustering

The accurate identification of the spatial domain is one of the important downstream tasks in spatial multi-omics data integration. The SpaMFG was compared with three mainstream spatial multi-omics integration methods, namely MISO, MOFA+, and SpatialGlue, on the human lymph node dataset. The human lymph node dataset contains spatial coordinates of transcriptome, proteome and spots. The spatial domain results provided manually by SpatialGlue (Long *et al.* 2024) were used as reference annotations to evaluate the accuracy of each method in spatial domain identification. These annotations provide biologically meaningful region labels for benchmarking. In this section of experiments, six supervised classification indicators of clustering were used as the indicators to measure the accuracy of spatial domain identification. The results are shown in Table 1. The SpaMFG achieved the best results on five of the six metrics, including NMI, AMI, V-measure, MI, and Homogeneity. For ARI, MISO obtained the highest score. Overall, SpaMFG showed competitive overall performance in spatial domain identification.

**Table 1:** Indicators for four spatial multi-omics data integration methods.^a^

Metrics	MOFA+	MISO	SpatialGlue	SpaMFG
ARI	0.298	**0.347**	0.297	0.316
NMI	0.401	0.404	0.393	**0.420**
AMI	0.399	0.403	0.391	**0.418**
V-Measure	0.401	0.404	0.393	**0.420**
MI	0.872	0.787	0.838	**0.887**
Homogeneity	0.517	0.467	0.497	**0.524**

a The data marked in bold are the best results for this row.

**Figure 2:**
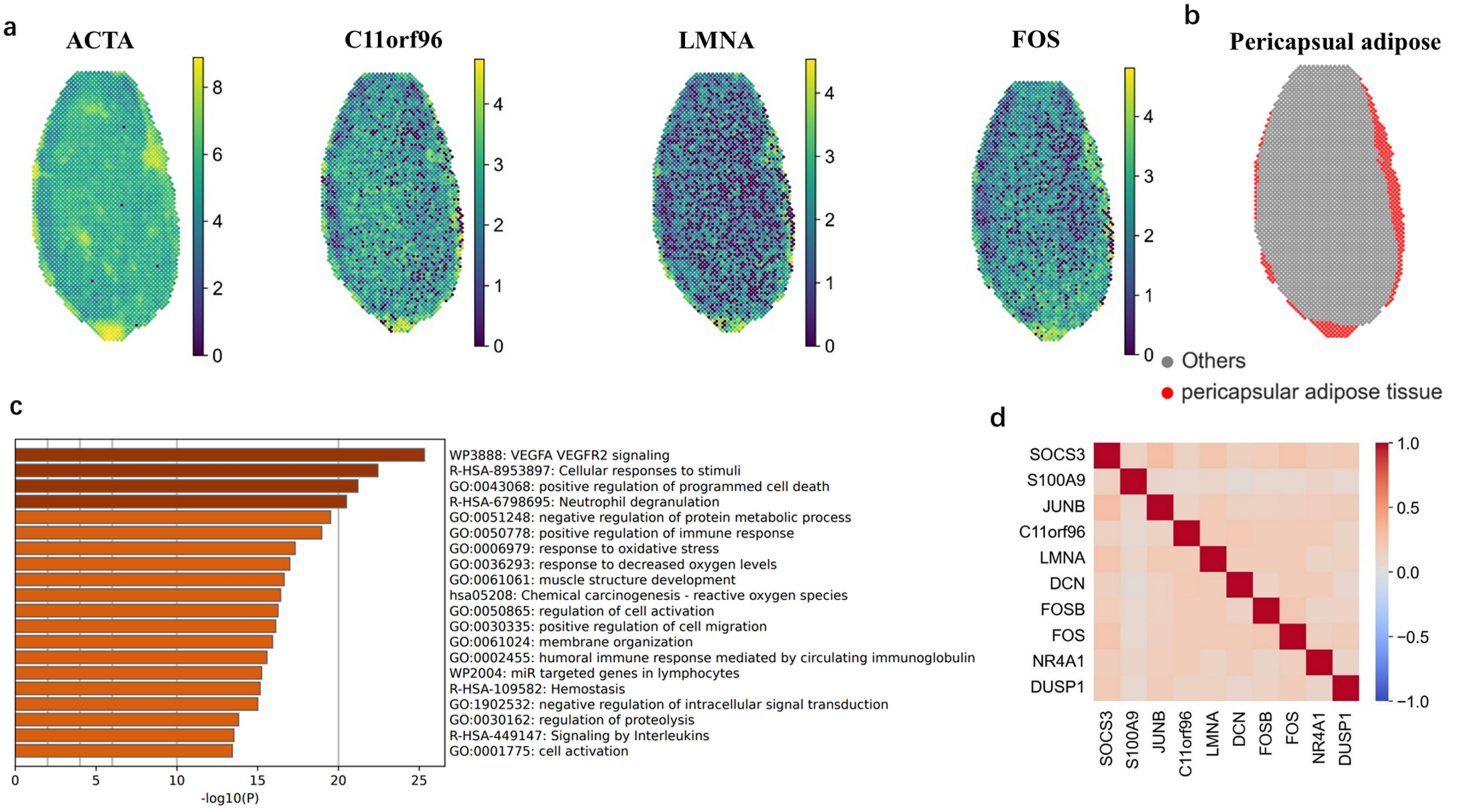
ACTA2-centered analysis on the human lymph node dataset. (a) Spatial expression of ACTA2-related genes. (b) Spatial distribution of the PAT region. (c) Enrichment analysis of the matched gene group. (d) Similarity heatmap of ACTA2-related genes. Color intensity indicates relative expression or similarity level.

In order to further verify the effectiveness of the feature grouping module, we selected the ACTA2 as a representative protein because it showed a clear spatially localized pattern in the pericapsular adipose tissue (PAT) region. We then found the corresponding gene group matched to it according to the results of the Hungarian algorithm, with the index being six. Subsequently, MetaScape (Zhou *et al.* 2019) was used to perform enrichment analysis on the sixth gene group, as shown in Fig. 2c. The functions of this gene set are mainly related to PAT. PAT is mainly located around the lymph node capsule and belongs to part of the lymphoid organs, and plays an important role in biological processes such as immune regulation, inflammatory response, and metabolic regulation (Pond 2005). From the figure, it can be seen that the functions involved in the sixth group of gene sets include immune response (GO:0050778), cell activation regulation (GO:0050865), humoral immune response mediated by circulating immunoglobulins (GO:0002455), etc. These functions are consistent with the mechanism by which PAT, through immune regulation and cell activation, plays a key role in the lymph nodes (Knight 2008). This result further supports that SpaMFG can effectively capture the biological features in spatial multi-omics data and reveals the functional association between specific proteins and matched gene groups.

To further validate the effectiveness of the feature grouping method based on spectral clustering in the integration of spatial multi-omics data, we analyze the spatial expression pattern of the ACTA2 protein. As shown in Fig. 2a, the expression of this protein is mainly concentrated in the PAT region. This result is consistent with the conclusion of the aforementioned enrichment analysis, further confirming that the feature grouping method can effectively identify feature groups with similar functions. Based on this, an in-depth study was conducted on the spatial expression pattern of ACTA2 protein. In the sixth gene set, genes with similar spatial expression patterns to ACTA2 were screened out, including FOS, LMNA, and C11orf96. Their spatial expression patterns are shown in Fig. 2a. The spatial expression patterns of these three genes are similar to that of ACTA2, indicating that the feature grouping method based on spectral clustering can extract features with similar spatial expression patterns from spatial multi-omics data. In order to quantify the correlations among genes within the feature group, the Spearman correlation (Spearman 1904) between all genes in the sixth group of genes and the ACTA2 protein was calculated, and the top 10 genes with the highest correlations were selected. Subsequently, the expression similarity among these 10 genes was calculated, as shown in Fig. 2d. The expression patterns of these 10 genes also exhibited a high degree-of-similarity, verifying the effectiveness of the feature grouping method based on spectral clustering in the integration of spatial multi-omics data.

### 3.2 Identification of human palatine tonsil spatial domains

**Figure 3:**
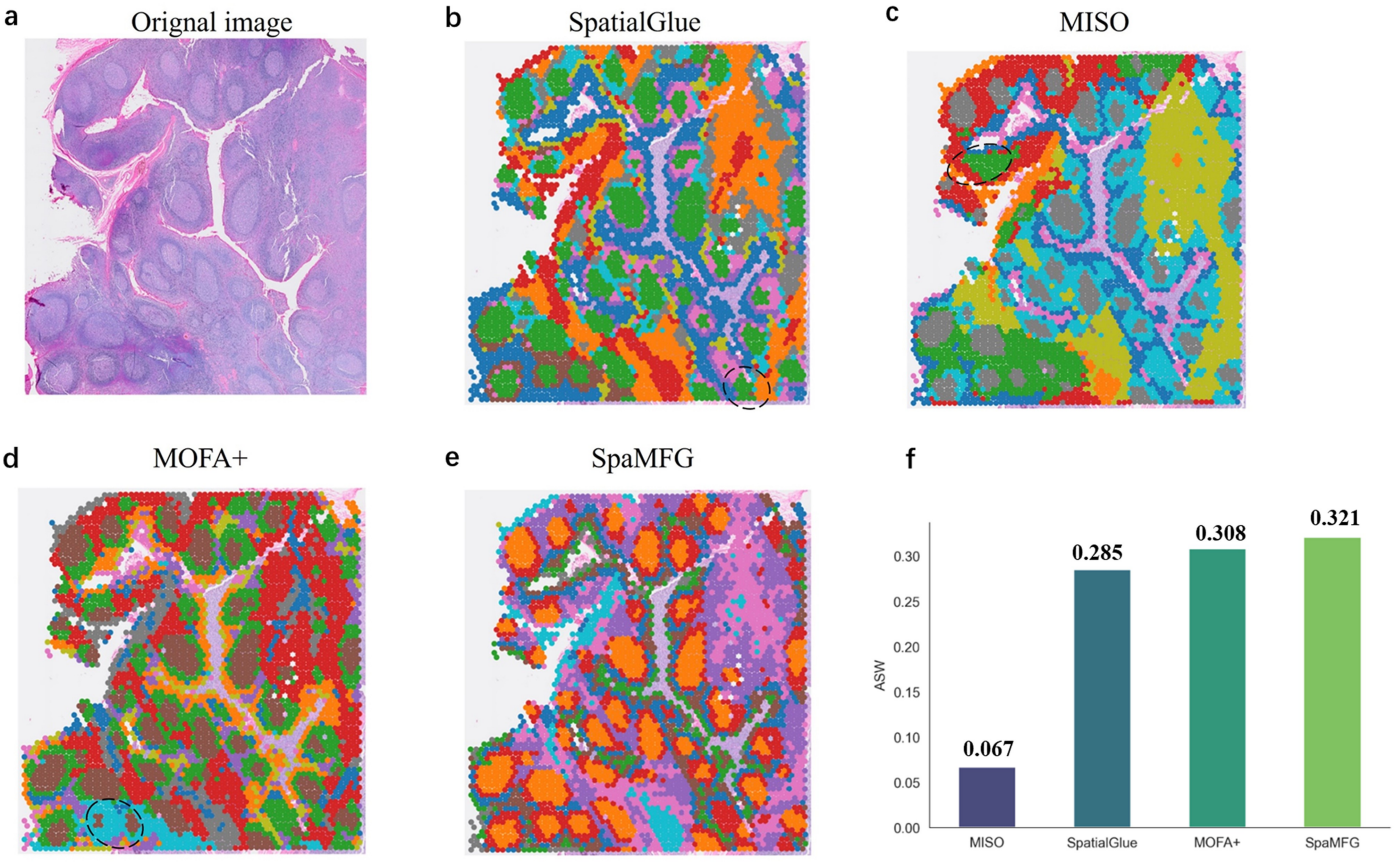
Spatial-domain identification on the human palatine tonsil dataset. (a) Histology-based germinal center annotation. (b–e) Results of the four compared methods. Different colors indicate distinct spatial domains. (f) ASW comparison of the four methods.

Spatial domain identification is one of the important downstream tasks in the integration of spatial multi-omics data, and its accuracy directly affects the biological interpretability of data analysis. To evaluate the performance of SpaMFG, we compared SpaMFG with three other methods on the human palatine tonsil dataset. The human tonsil dataset covers spatial transcriptomics, spatial proteomics, and histological imaging data. MISO used transcriptomic, proteomic, and histological image data, whereas SpaMFG used transcriptomic, proteomic, and spatial coordinate data. Therefore, this comparison should be interpreted with this input difference in mind. Figure 3 shows the germinal center regions identified by high-resolution histological images, where each circle in the histological images represents the germinal center. SpaMFG demonstrated high accuracy in identifying germinal centers, and the size and location of the identified germinal centers were very close to those in the original tissue images. Without using histological images, SpaMFG still identified germinal center regions close to the histology-based annotation. Although SpatialGlue could also identify germinal centers, it had omissions in some edge areas, resulting in some germinal center boundaries not being accurately captured. The missing parts have been circled in Fig. 3a–e. MOFA+ could also identify germinal centers, but its recognition results in some areas deviated from the original histological images, and these areas have also been marked in the figure, indicating that its resolution ability in certain spatial structures was limited. MISO also had similar problems in identifying germinal centers, indicating that its accuracy in spatial domain recognition was limited. To quantitatively evaluate the integration effect of the four methods in the spatial domain recognition task, average silhouette width (ASW) was used as the quantitative evaluation index. The results are shown in Fig. 3f. On this dataset, SpaMFG had the highest ASW value, MOFA+ had the second-highest, and the MISO method had the lowest score in the ASW index. This result suggests that SpaMFG can effectively identify germinal center regions using molecular and spatial information.

**Figure 4:**
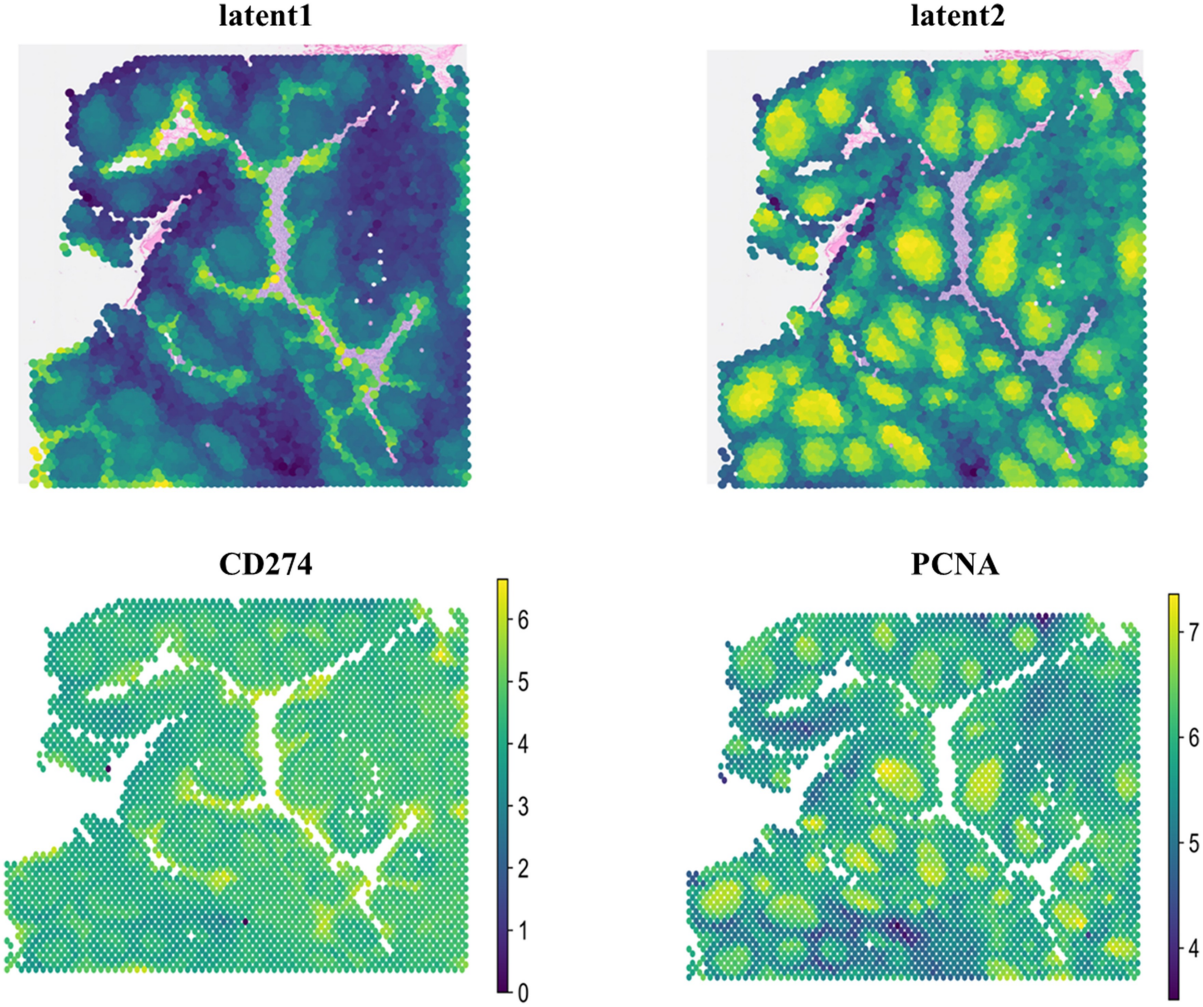
SpaMFG embeddings and representative protein expression patterns on the human palatine tonsil dataset. Color intensity indicates latent representation values or relative protein expression levels.

To further verify the interpretability of the integrated results of SpaMFG, this paper visualizes the spatial distribution of the first two dimensions of the potential representation of SpaMFG. As shown in Fig. 4, the spatial distribution of the first dimension embedded is highly similar to the expression pattern of CD274 protein, while the potential representation space distribution of the second dimension is highly consistent with the expression pattern of PCNA protein. Among them, PCNA protein is a known protein that has been verified by biological experiments and shows a significantly elevated expression in the germinal center (Holmes *et al.* 2020). The second dimension of SpaMFG’s potential representation is consistent with its expression pattern, indicating that this dimension can accurately capture the important biological characteristics of the germinal center region, and demonstrates the strong biological interpretability of SpaMFG. In conclusion, the comparative analysis and quantitative evaluation **s**upport the effectiveness of SpaMFG on the human palatine tonsil dataset.

### 3.3 Identification of mouse spleen spatial domain

**Figure 5:**
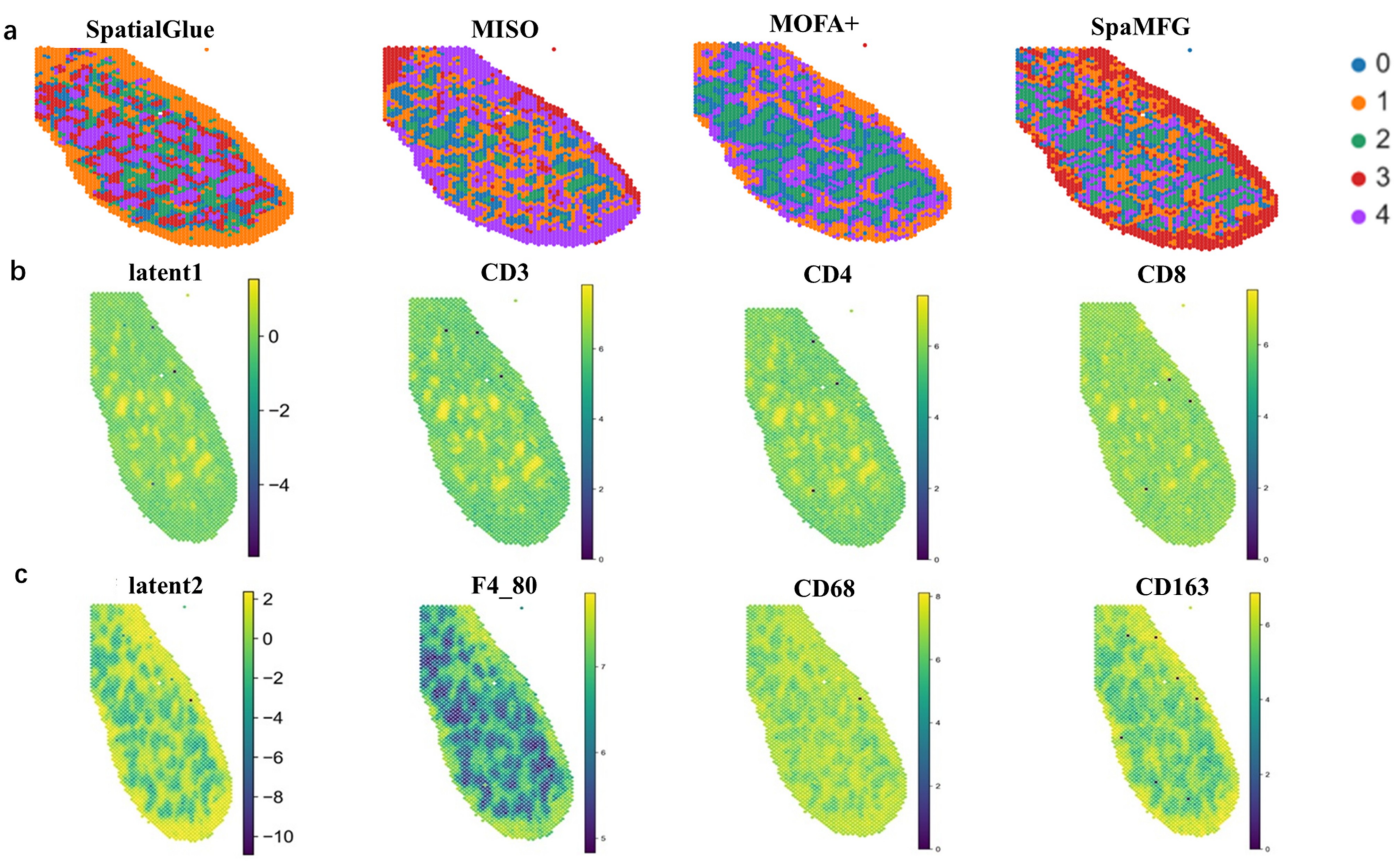
Spatial domain identification results on the mouse spleen dataset. (a) Spatial domain recognition results of the four compared methods. (b) Visualization of the first SpaMFG latent dimension and spatial expression patterns of CD3, CD4, and CD8. (c) Visualization of the second SpaMFG latent dimension and spatial expression patterns of F4-80, CD68, and CD163.

This experiment utilized a mouse spleen dataset generated by SPOTS technology, which contained transcriptome, protein, and spatial coordinate data. During the sequencing process, this dataset identified B cells, T cells, and macrophages using protein markers, providing biological prior knowledge for spatial domain identification, and annotated the distribution of different immune cell subsets in the spleen based on marker proteins and differentially expressed genes. To evaluate the performance of SpaMFG on this dataset, the spatial domain identification results of four methods on this dataset were presented, as shown in Fig. 5a. Three subgroups of macrophages Compared with the other three methods, the spatial domain identification results of SpaMFG were more similar to the annotation results of marker proteins, indicating the effectiveness of SpaMFG.

To quantitatively evaluate the spatial domain identification results of SpaMFG, MISO, MOFA+, and SpatialGlue methods on this dataset, ASW was used as an evaluation index. The results are shown in Supplementary Fig. S1, available as supplementary data at *Bioinformatics* online. SpaMFG achieved the highest ASW value on this dataset, indicating the best spatial domain identification results. MOFA+ was second, and MISO was the lowest. This shows that SpaMFG achieves strong performance in spatial domain identification and further supports its stability and robustness in spatial multi-omics data integration.

To further analyze the interpretability of the integrated results of SpaMFG, the distribution results of the first two dimensions of the latent space and the expression patterns of known proteins were presented. The results are shown in Fig. 5b and c. The spatial distribution represented by the first dimension of the latent representation of SpaMFG is highly similar to the expression patterns of CD3 protein, CD4 protein, and CD8 protein. Among them, CD3 is a typical marker protein of T cells (Cesta 2006), and the distribution of T cells in the white pulp of the spleen mainly gathers in small clusters. The first dimension of the latent representation of SpaMFG can accurately capture the spatial distribution pattern of T cells, indicating that its integrated results have a high biological interpretability. The spatial distribution represented by the second dimension of the latent representation is very similar to the expression patterns of F4-80, F4-80, and CD163 proteins. Among them (Lin *et al.* 2005). The second dimension of the latent representation of SpaMFG accurately matches the spatial expression pattern of macrophages, indicating that it not only can integrate spatial multi-omics data but also can retain the spatial distribution information of different types of immune cells in the spleen in the latent representation, further enhancing the interpretability of the model.

### 3.4 Identification of mouse thymus spatial domain

Finally, to further verify the robustness of the SpaMFG, it was applied to the mouse thymus dataset. The thymus is a small gland surrounded by fibrous and collagenous tissues, consisting of a connected connective tissue island fissure and two separated lobes (Pearse 2006). Each lobe is roughly divided into the central medulla and the outer cortex layer. In this experiment, the mouse thymus dataset generated by the Stereo-CITE-seq technology was used. This technology can capture spatial transcriptomic and spatial proteomic data at the subcellular resolution, thereby providing more refined spatial resolution capabilities.

On this dataset, the spatial domain recognition performance of the four methods, namely MOFA+, SpatialGlue, MISO, and SpaMFG, was compared and analyzed. The input of all methods was transcriptomic, proteomic data, and spatial coordinate information. The spatial domain recognition results of the four methods are shown in Supplementary Fig. S2, available as supplementary data at *Bioinformatics* online. Among them, MISO failed to identify the cortex and medulla regions within the thymus, and the coherence of the spatial domains was poor. This result was also reflected in the ASW score, as shown in Supplementary Fig. S3, available as supplementary data at *Bioinformatics* online. The ASW score of MISO was the lowest. MOFA+ and SpatialGlue relatively better captured the cortico-medullary junction and the inner, middle, and outer cortex (clusters 2–5). MOFA+ failed to accurately identify some regions, indicating that its resolution ability in certain spatial structures is insufficient. SpatialGlue could identify this region, but its ASW score was lower than SpaMFG. Overall, SpaMFG scored the highest in ASW score, indicating strong performance on this dataset.

**Figure 6:**
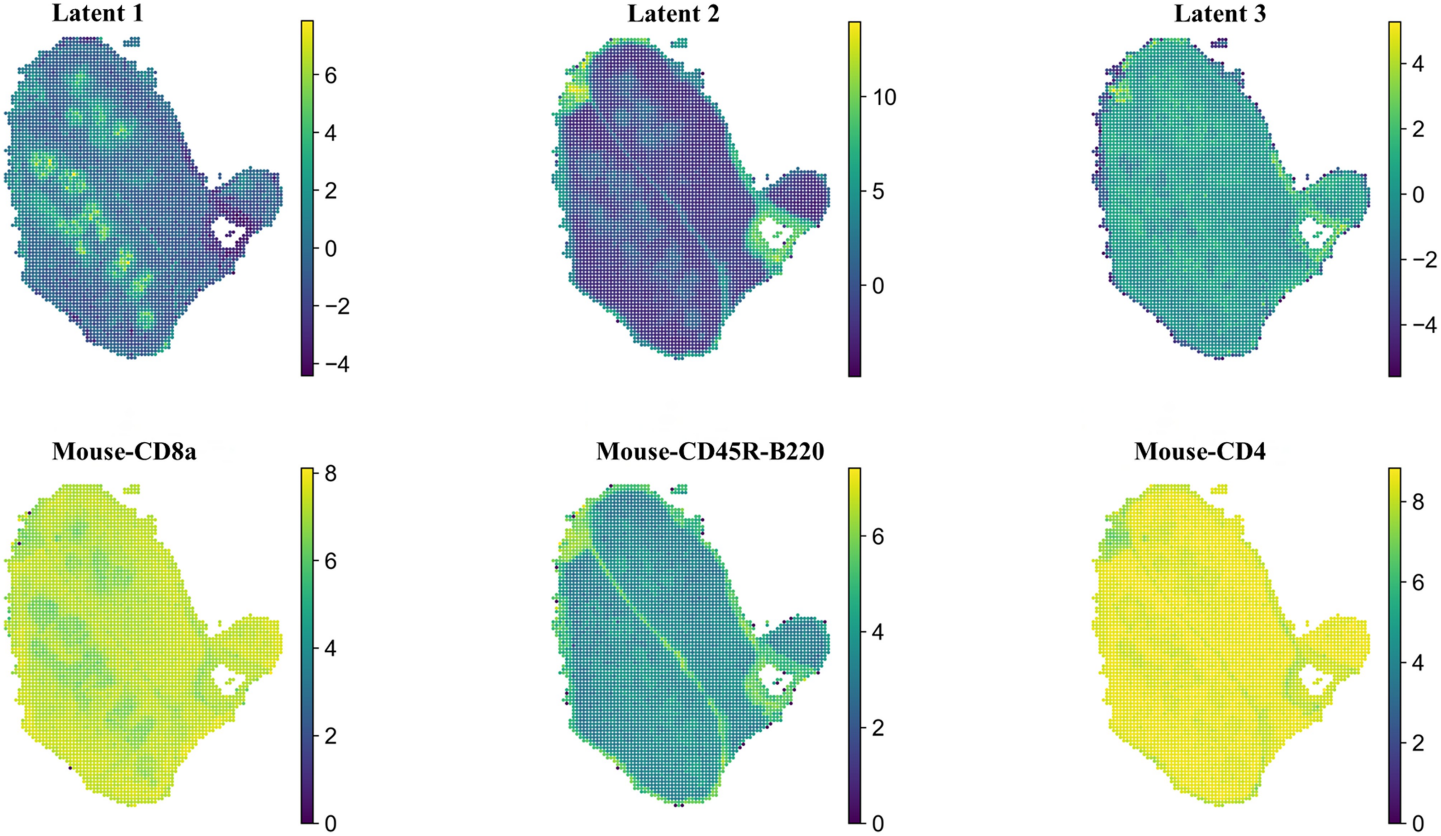
Visualization results of the three-dimensional embedding of SpaMFG and the spatial expression distribution of the three proteins.

Similarly, to further validate the biological interpretability of the SpaMFG integration results, the spatial distribution of the top three-dimensional latent representations of SpaMFG was demonstrated, and it was compared with the expression patterns of known proteins. As shown in Fig. 6, the spatial distribution of the first-dimensional latent representation was highly consistent with the expression pattern of the CD8a protein, which is mainly expressed in the cortical and medullary regions. The spatial distribution of the second-dimensional latent representation was similar to that of the CD45R-B220 protein, and the spatial distribution of the third-dimensional latent representation was consistent with that of the CD4 protein. These results indicate that the latent representations of the SpaMFG method can accurately match the spatial organizational features in the mouse thymus dataset, verifying the interpretability and biological rationality of the model on the mouse thymus dataset.

## 4 Discussion

The SpaMFG method, its outstanding performance proves the effectiveness of the feature grouping method based on spectral clustering in spatial domain identification. However, SpaMFG still has room for further improvement. Firstly, SpaMFG is mainly used for integrating spatial transcriptomics and proteomics, and can be extended to metabolomics and histological images, etc. in the future; Secondly, with the continuous growth of the scale of spatial multi-omics data, algorithms based on spectral clustering for memory. The demand for computing resources is relatively high. In the future, attempts can be made to introduce distributed computing frameworks, etc., thereby improving computing efficiency. Finally, the current feature group matching mainly relies on data-driven linear allocation methods. Because the proteomic layer usually contains fewer measured features, some transcriptomic spatial patterns may not be fully represented at the protein level. Therefore, SpaMFG may emphasize shared cross-omics spatial signals and underrepresent transcriptome-specific patterns. In the future, prior feature function information can be integrated, such as introducing KEGG pathway analysis, constructing protein-protein interaction networks, and introducing pathway contribution weights in the cost matrix to enhance the biological interpretability of the matched feature groups. Future work could also allow unmatched transcriptomic groups or introduce modality-specific residual representations to better preserve transcriptome-specific spatial variation.

## 5 Conclusions

This method takes into account the importance of spatial coordinate information in identifying the spatial domain. The main steps include feature grouping, feature group matching and feature group integration. Firstly, based on spectral clustering, identify the features of each omics with similar spatial expression patterns. Then, spatial weights and the Hungarian algorithm are introduced to match the optimal feature group. Finally, MOFA is used to integrate each matching feature group and concatenate the results to obtain the final embedded representation. This paper conducted comparative experiments with three methods on the dataset of human lymph nodes with real annotations. The results showed that SpaMFG significantly improved the spatial domain recognition performance of spatial multi-omics data, verifying the effectiveness of SpaMFG. Subsequent experimental results on datasets of human tonsils, mouse spleens and mouse thymus show that SpaMFG can effectively identify the spatial domain distribution of complex datasets and reveal the functional associations among characteristic groups, providing important information for the study of tissue structure and its function.

## Supplementary Material

btag457_Supplementary_Data

## Data Availability

The source code and datasets used in this study are freely available at: https://github.com/LiangYu-Xidian/SpaMFG.
