# Supplementary material for: SpaMFG: a spatial multi-omics integration method based on feature grouping
Source: Bioinformatics. 2026 Jun 30;42(7):btag457. doi: 10.1093/bioinformatics/btag457 (PMC13360273; doi:10.1093/bioinformatics/btag457)
Supplement: btag457_Supplementary_Data [file btag457_supplementary_data.docx]

SpaMFG：a Spatial Multi-omics Integration Method based on Feature Grouping


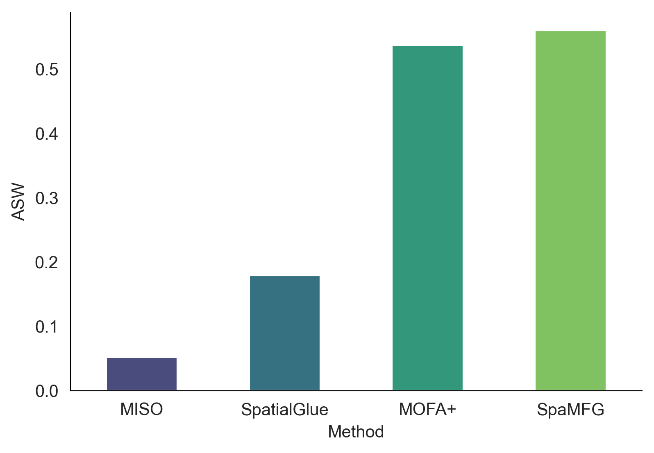


Fig. S1 The ASW comparison results of four methods on the Mouse Spleen dataset.


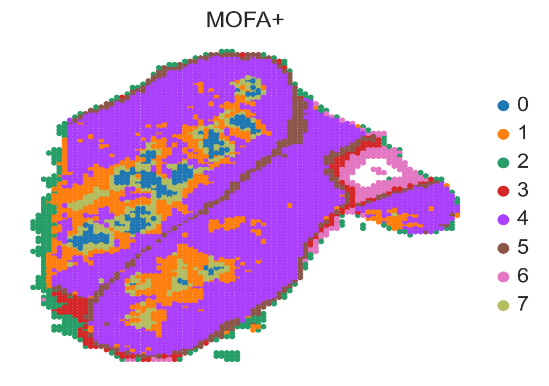

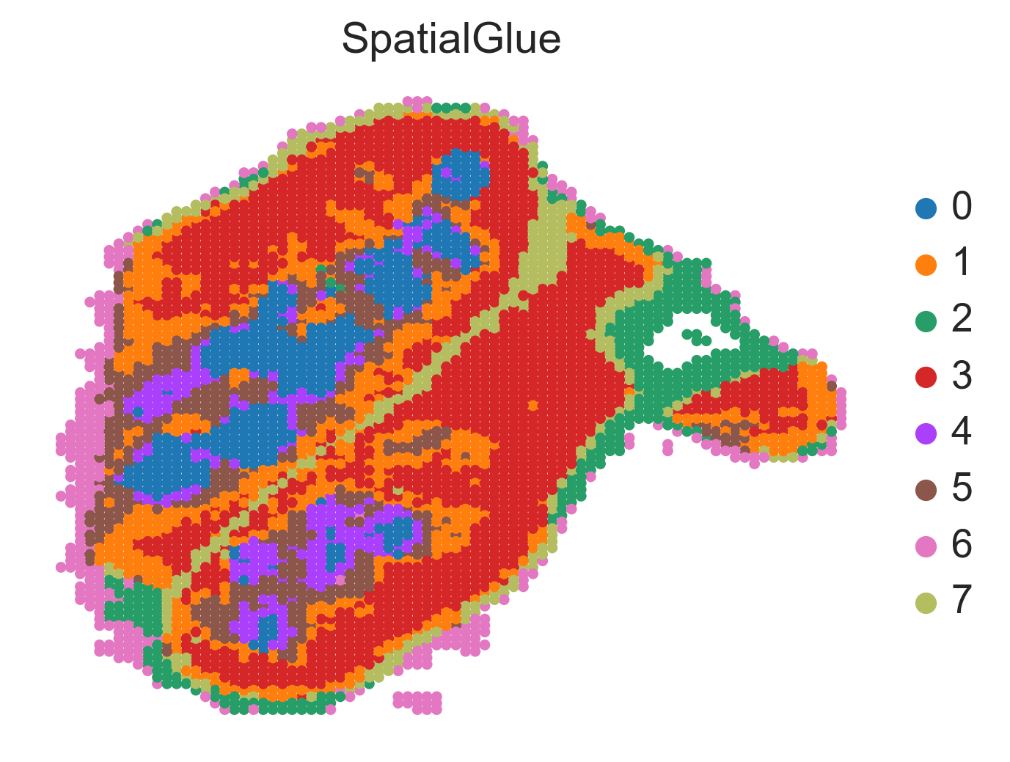

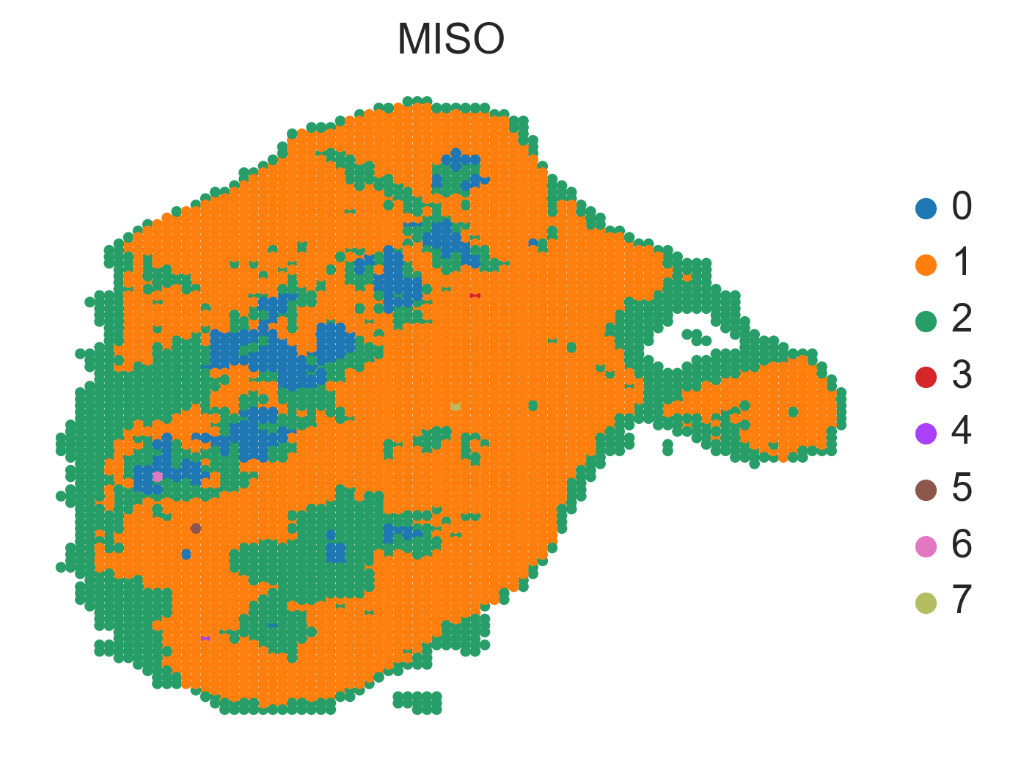

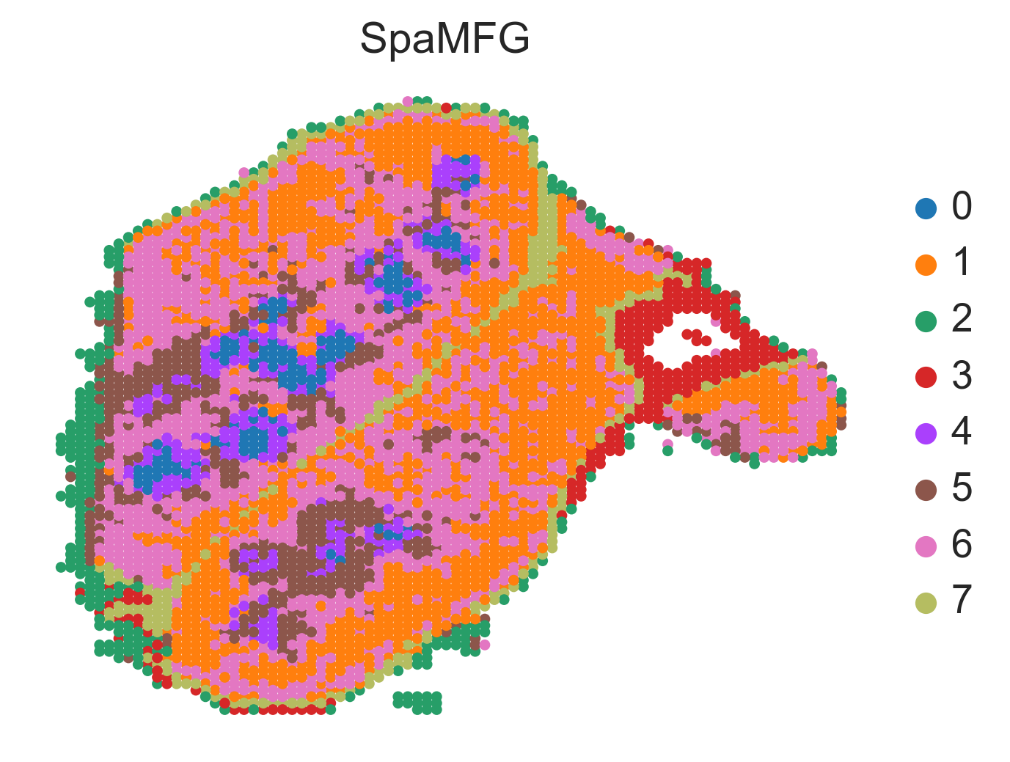


Fig. S2 The results of spatial domain recognition of Mouse Thymus data.


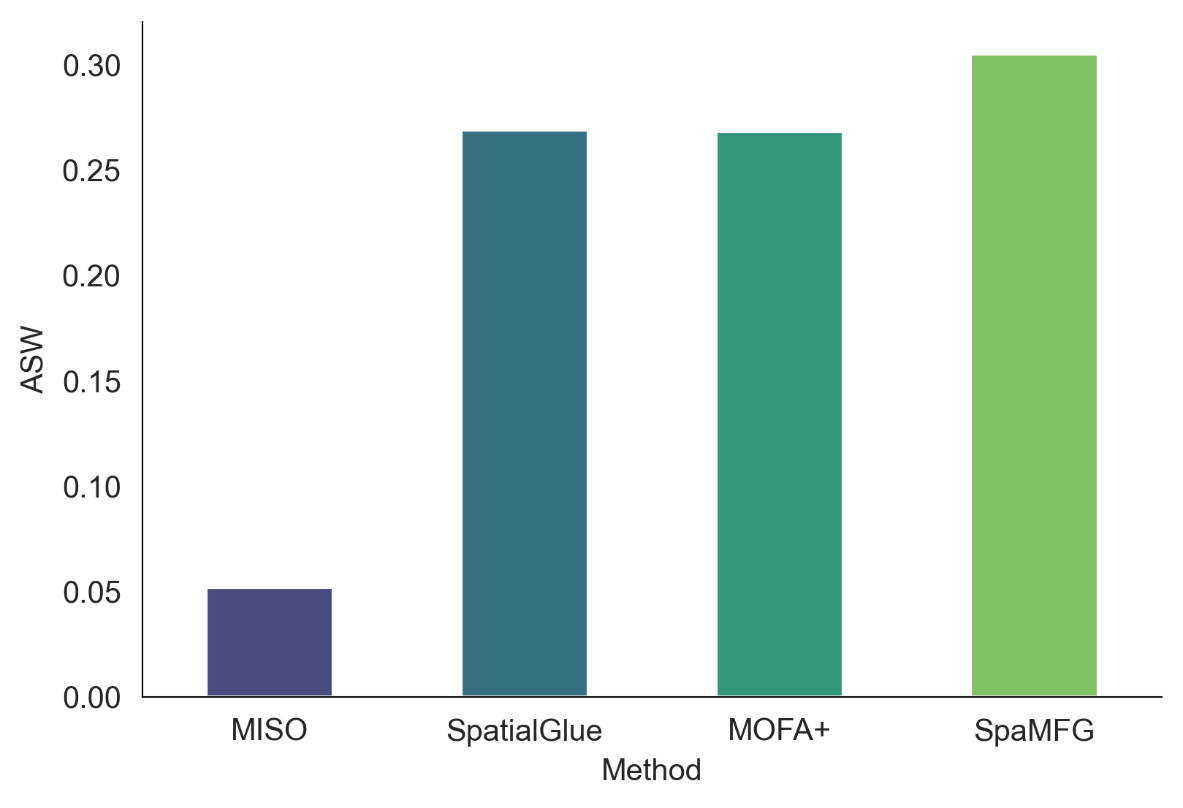


Fig. S3 The ASW comparison results of four methods on the Mouse Thymus dataset.
